# Predictive Neuronal Adaptation as a Basis for Consciousness

**DOI:** 10.3389/fnsys.2021.767461

**Published:** 2022-01-11

**Authors:** Artur Luczak, Yoshimasa Kubo

**Affiliations:** Canadian Center for Behavioural Neuroscience, University of Lethbridge, Lethbridge, AB, Canada

**Keywords:** brain-inspired artificial neuronal networks, neuronal adaptation, theory of consciousness, biological learning algorithms, anesthesia

## Abstract

Being able to correctly predict the future and to adjust own actions accordingly can offer a great survival advantage. In fact, this could be the main reason why brains evolved. Consciousness, the most mysterious feature of brain activity, also seems to be related to predicting the future and detecting surprise: a mismatch between actual and predicted situation. Similarly at a single neuron level, predicting future activity and adapting synaptic inputs accordingly was shown to be the best strategy to maximize the metabolic energy for a neuron. Following on these ideas, here we examined if surprise minimization by single neurons could be a basis for consciousness. First, we showed in simulations that as a neural network learns a new task, then the surprise within neurons (defined as the difference between actual and expected activity) changes similarly to the consciousness of skills in humans. Moreover, implementing adaptation of neuronal activity to minimize surprise at fast time scales (tens of milliseconds) resulted in improved network performance. This improvement is likely because adapting activity based on the internal predictive model allows each neuron to make a more “educated” response to stimuli. Based on those results, we propose that the neuronal predictive adaptation to minimize surprise could be a basic building block of conscious processing. Such adaptation allows neurons to exchange information about own predictions and thus to build more complex predictive models. To be precise, we provide an equation to quantify consciousness as the amount of surprise minus the size of the adaptation error. Since neuronal adaptation can be studied experimentally, this can allow testing directly our hypothesis. Specifically, we postulate that any substance affecting neuronal adaptation will also affect consciousness. Interestingly, our predictive adaptation hypothesis is consistent with multiple ideas presented previously in diverse theories of consciousness, such as global workspace theory, integrated information, attention schema theory, and predictive processing framework. In summary, we present a theoretical, computational, and experimental support for the hypothesis that neuronal adaptation is a possible biological mechanism of conscious processing, and we discuss how this could provide a step toward a unified theory of consciousness.

## Introduction

“How does the brain work? Gather enough philosophers, psychologists, and neuroscientists together (ideally with a few mathematicians and clinicians added to the mix), and I guarantee that a group will rapidly form to advocate for one answer in particular: that the brain is a prediction machine” (Seth, 2020). Predictive processing was also suggested to be one of the most promising approaches to understand consciousness (Yufik and Friston, 2016; Hohwy and Seth, 2020). Nevertheless, it is still unclear how predictive processing could be implemented in the brain (Lillicrap et al., 2020), as most of the proposed algorithms require a precise network configuration (Rao and Ballard, 2005; Bastos et al., 2012; Whittington and Bogacz, 2017), which could be difficult to achieve, considering variability in neuronal circuits (y Cajal, 1911).

To address this problem, we proposed that single neurons can internally calculate predictions, which eliminates requirement of precise neuronal circuits (Luczak et al., 2022). Biological neurons have a variety of intracellular processes suitable for implementing predictions (Gutfreund et al., 1995; Stuart and Sakmann, 1995; Koch et al., 1996; Larkum et al., 1999; Ha and Cheong, 2017). The most likely candidate for realizing predictive neuronal mechanism appears to be calcium signaling (Bittner et al., 2017). For instance, when a neuron is activated, it leads to a higher level of somatic calcium lasting for tens of ms (Ali and Kwan, 2019). As neuron activity is correlated with its past activity within tens of ms (Harris et al., 2003; Luczak et al., 2004), thus, lasting increase in calcium concentration may serve as a simple predictive signal that a higher level of follow up activity is expected. Notably, basic properties of neurons are highly conserved throughout evolution (Kandel et al., 2000; Gomez et al., 2001; Roberts and Glanzman, 2003), therefore a single neuron with a predictive mechanism could provide an elementary unit to build predictive brains for diverse groups of animals.

**FIGURE 1:**
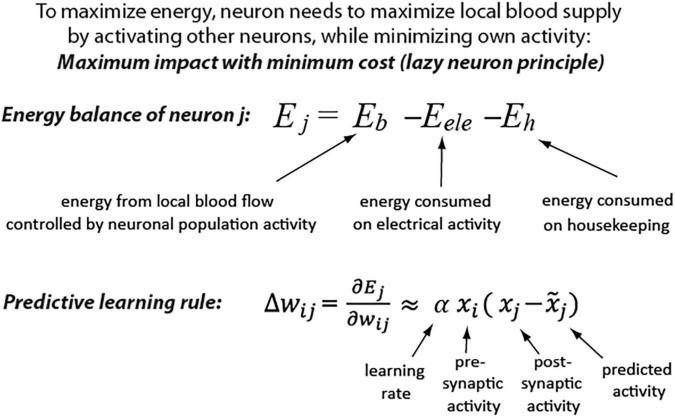
Maximizing neuron metabolic energy leads to predictive synaptic learning rule (see Supplementary Material for derivation details).

This idea is further supported by a theoretical derivation showing that the predictive learning rule provides an optimal strategy for maximizing metabolic energy of a neuron. The details of derivation are described in a study (Luczak et al., 2022) and a summary is depicted in Figure 1. Shortly, *E*_*b*_ represents energy received from blood vessels in the form of glucose and oxygen, which is a non-linear function of local neuronal population activity, including the considered neuron *j* activity (*x*_*j*_) (Devor et al., 2003; Sokoloff, 2008). The *E*_*ele*_ represents the energy consumed by a neuron for electrical activity, which is mostly a function of the presynaptic activity (*x*_*i*_) and respective synaptic weights (*w*_*ij*_) (Harris et al., 2012). A neuron also consumes energy on housekeeping functions, which could be represented by a constant *E*_*h*_. As described in a study (Luczak et al., 2022), this formulation shows that to maximize energy balance, a neuron has to minimize its electrical activity (be active as little as possible), but at the same time, it should maximize its impact on other neurons’ activities to increase blood supply (be active as much as possible). Thus, weights must be adjusted to strike a balance between two opposing demands: maximizing the neuron’s downstream impact and minimizing its own activity (cost). This energy objective of a cell could be paraphrased as the “*lazy neuron principle: maximum impact with minimum activity.*” We can calculate such required changes in synaptic weights (Δ*w)* that will maximize neuron’s energy (*E*_*j*_) by using gradient ascent method [for derivation see Supplementary Material or (Luczak et al., 2022)]. As a result, we found that maximizing future energy balance by a neuron leads to a predictive learning rule, where a neuron adjusts its synaptic weights to minimize surprise [i.e., the difference between actual (*x*_*j*_) and predicted activity (${\tilde{x}}_{j})$].

Interestingly, this derived learning rule was shown to be a generalization of Hebbian-based rules and other biologically inspired learning algorithms, such as predictive coding and temporal difference learning (Luczak et al., 2022). For example, when ${\tilde{x}}_{j}=0$ in our predictive learning rule (i.e., when a neuron does not make any prediction), then we obtain Hebb’s rule: Δ*w*_*ij*_ = α*x*_*i*_*x*_*j*_, a.k.a. “cells that fire together, wire together”(Hebb, 1949). Moreover, our model belongs to the category of energy-based models, for which it was shown that synaptic update rules are consistent with spike-timing-dependent plasticity (Bengio et al., 2017). Thus, this predictive learning rule may provide a theoretical connection between multiple brain-inspired algorithms and may offer a step toward development of a unified theory of neuronal learning.

The goal of this paper is to show that the properties ascribed to consciousness could be explained in terms of predictive learning within single neurons. For that, first, we will implement a predictive learning rule in an artificial neural network, and then we will use those simulation results together with biological evidence to propose a predictive neuronal adaptation theory of consciousness.

## Methods

### Implementation of a Predictive Learning Rule in a Neural Network

**FIGURE 2:**
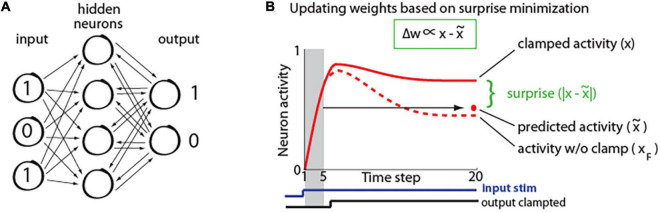
**(A)** Simplified schematic of our recurrent network architecture. For visualization, only a small subset of neurons is shown. **(B)** Illustration of neuron activity in response to a stimulus. Initially the network receives only the input signal (bottom blue trace), but after 8 steps, the output signal is also presented (a.k.a. clamped phase; bottom black trace). The red dot represents steady-state activity which was predicted from initial activity (in shaded region). The dashed line shows activity of the same neuron in response to the same stimulus, if the output would not be clamped (*x*_*F*_; a.k.a. free phase), which neuron “wants” to predict. Green insert: synaptic weights (w) are adjusted in proportion (∝) to the difference between steady-state activity in clamped phase (*x*) and predicted activity ($\tilde{x}$) [adopted from Luczak et al. (2022)].

To study how properties of predictive learning rule may relate to consciousness processes, we created a recurrent neural network. It had 420 input units, 50 hidden units, and 10 output units as illustrated in Figure 2A. The network was trained on a hand-written digit recognition task MNIST (LeCun et al., 1998), with 21 × 20 pixels from center of each image given as input to the network. The details of network training are described in a study (Luczak et al., 2022). First, network is presented with only an input signal and the activity starts propagating throughout the network until it converges to a steady-state, when the neurons’ activity stops changing, as depicted in Figure 2B. This is repeated for 1,600 randomly chosen stimuli. During this phase, we also trained a linear model to predict the steady-state activity. Specifically, for each individual neuron, the activity during the five initial time steps (*x*_(1)_,…,*x*_(5)_) was used to predict its steady-state activity at time step 20: *x*_*(20)*_, such that: $x_{\left(20\right)}\,\text{≈}\,\tilde{x}=\lambda_{\left(1\right)}*x_{\left(1\right)},+\cdots +\lambda_{\left(5\right)}*x_{\left(5\right)}+b$, where $\tilde{x}$ denotes predicted activity, λ and *b* correspond to coefficients and offset terms of the least-squared model, and the terms in brackets correspond to time steps (Figure 2B). Next, a new set of 400 stimuli was used, where from step 8, the network output was clamped at values corresponding to image class (teaching signal). For example, if the image of number 5 was presented, then the value of the 5th output neuron was set to “1,” and the values of the other 9 output neurons was set to “0,” and network was allowed to settle to the steady-state. This steady-state was then compared with predicted steady-state activity, which was calculated using the above least-squared model. Subsequently, for each neuron, the weights were updated based on the difference between the actual (*x*_*j*_) and its predicted activity (${\tilde{x}}_{j}$) in proportion to each input contribution (*x*_*i*_), as prescribed by the predictive learning rule in Figure 1 (Matlab code for a sample network with our predictive learning rule is provided in Supplementary Material).

## Results

### Neuronal Surprise Reproduces Stages of Skill Consciousness

**FIGURE 3:**
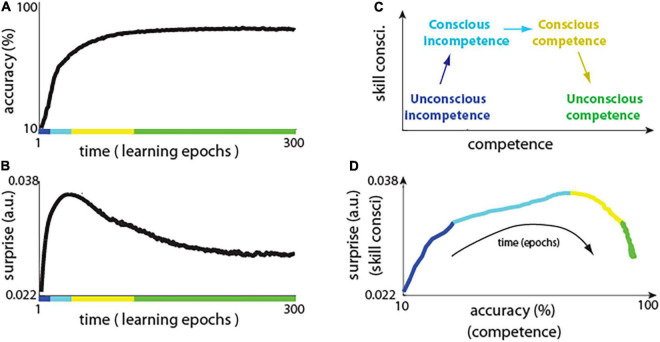
Neuronal surprise in network with predictive learning rule. **(A)** Learning curve showing accuracy of the network across learning epochs. Colors indicate conceivable extents of stages of “conscious competence” shown in panel **(C)**. **(B)** Change in neuronal surprise (|actual – predicted activity|) averaged over all neurons (see main text for details). **(C)** Diagram of the four stages of “conscious competence” during skill learning. **(D)** Neuronal surprise (proxy of skill consci.) vs accuracy (a.k.a. competence) during network learning. This is replotting data from panels **(A)** vs **(B)**, where each point corresponds to a single time epoch. Note that although for example, “unconscious competence” (marked in green) spans over 150 epochs in panels **(A)** and **(B)**, in panel **(C)** those points are “compressed” as there is little change in accuracy, and in surprise during those epochs.

The network using predictive learning rule showed a typical learning curve, with rapid improvement in performance in the first few training epochs, and with plateauing performance during later training epochs (Figure 3A). Notably, this shape of learning curve is also typical for skill-learning in humans, where, initially at the novice level, there are fast improvements, and it takes exponentially more time to improve skills at, for example, elite athlete level (Newell and Rosenbloom, 1981). However, what is new and interesting here, is how a surprise (i.e., the difference between actual and predicted activity) evolved during network training (Figure 3B) and how it compares to the stages of “skill consciousness,” as explained below.

It was observed that learning involves the four stages of “conscious competence” (Broadwell, 1969; Das and Biswas, 2018; Figure 3C): (1) Unconscious incompetence – where individual does not know what he/she doesn’t know, and, thus, that individual is not aware of his/her own knowledge deficiencies (e.g., foreigner may not know about certain local traffic regulations); (2) Conscious incompetence – where the individual recognizes his/her own lack of knowledge or skills but does not have those skills (e.g., a car passenger who does not know how to drive); (3) Conscious competence – where the individual develops skills but using it requires conscious effort (e.g., beginner car driver); (4) Unconscious competence - where due to extensive practice, the individual can perform learned tasks on “autopilot” (e.g., driving car on the same route every day).

Here we illustrate how the above stages of conscious competence could be recapitulated by the network with our predictive learning rule. We used the neuronal surprise as a proxy measure of consciousness, which is motivated by previous theoretical (Friston, 2018; Waade et al., 2020) and experimental work (Babiloni et al., 2006; Del Cul et al., 2007), which will be discussed in later sections. We calculated the surprise for each neuron *j* as: < |$x_{j}-{\tilde{x}}_{j}$|>, where |…| denotes absolute value, and < … > denotes average across all 400 images presented in a single training epoch. The neuronal surprise was defined as mean surprise across all of neurons. To better illustrate the network behavior, we also plotted accuracy (a.k.a. competence) vs surprise (a proxy of consciousness) (Figure 3D; model details and code to reproduce presented figures are included in Supplementary Material). Initially, when the network was presented with an input image, the neurons in the hidden layer could almost perfectly predict what will be the steady-state activity after the output units are clamped (Figure 3B, first few epochs). This is because the network starts with random connections and the signal coming from 10 output units is relatively week in comparison to the signal coming to the hidden layer from a much larger number of input units: 420. Thus, the steady-state activity, which neurons learn to predict when only the input image is presented, is not much different from the steady-state activity when input image is presented together with clamped outputs. This is like the “unconscious incompetence” stage, as the network is almost completely “not aware” of the teaching (clamped) signal (Figure 3D, blue line). However, as the activity of the output neurons is mostly correlated with any discrepancy between the actual and the predicted activity in hidden layer neurons, thus, the synaptic weights from output neurons are most strongly modified. Consequently, as the learning progresses, the hidden neurons are more and more affected by the output units, and their surprise: the discrepancy between actual and predicted activity, increases. This is analogous to the “conscious incompetence,” where the network becomes “aware” of the clamped teaching signal, but the network has not yet learned how to classify images correctly (Figure 3D, light blue). In result, as magnitude of surprise |$x_{j}-{\tilde{x}}_{j}$| increases, then other synaptic weights also start changing more, as prescribed by the predictive learning rule in Figure 1. Those synaptic updates made the activity driven by the input image, closer to the desired activity as represented by the clamped output units. This could be characterized as “conscious competence,” where the surprise signal allows the network to learn and to become more competent on that task (Figure 3D, yellow line). Finally, as network learns to predict the image class with high accuracy, then the surprise (the difference between predicted and clamped teaching signal) is diminishing, which is analogous to an expert who achieved “unconscious competence” (Figure 3D, green line). This, that the neuronal surprise recapitulates the stages of conscious competence, by first increasing and then decreasing during learning, was a general phenomenon across different datasets and across diverse network architectures (Supplementary Figure 1).

### Surprise Reduction by Neuronal Adaptation

Derivation of the predictive learning rule in Figure 1 shows that the best strategy for a cell to maximize metabolic energy is by adjusting its synaptic weights to minimize surprise: |$x-\tilde{x}|$. However, this change in surprise does not need to take minutes or hours, as typically required for structural synaptic modification to occur (Xu et al., 2009). Neurons have adaptation mechanisms, which could serve to reduce surprise at a much faster time scale of tens of ms (Whitmire and Stanley, 2016).

**FIGURE 4:**
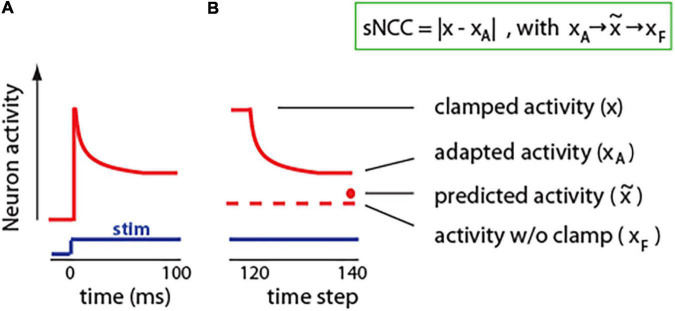
Adaptation in neurons and in our model. **(A)** Cartoon illustration of typical neuronal adaptation in response to constant stimuli. Note that after the initial increase, neuronal activity moves closer toward the activity level without stimulus. **(B)** Similarly, in our model, adaptation shifts neuronal activity toward activity level without clamp (predicted activity). Insert: We propose to define a single-neuron correlate of consciousness (sNCC) as neuronal adaptation. Arrows denote that activity adapts toward predicted activity, which approximates activity without a clamp.

Neural adaptation is a ubiquitous phenomenon that can be observed in neurons in the periphery, as well as in the central nervous system; in vertebrates, as well as in invertebrates (Whitmire and Stanley, 2016; Benda, 2021). Neuronal adaptation can be defined as the change in activity in response to the same stimulus. The stimulus can be a current injection into a single neuron or a sensory input like sound, light, or whisker stimulation. Usually, neuron activity adapts in exponential-like fashion, with rapid adaptation at the beginning, and then later plateauing at a steady-state value (Figure 4A). Typically, neuronal adaptation is shown as the decrease in activity in response to excitatory stimuli. However, neurons can also adapt by increasing its spiking ability when inhibitory stimulus is presented; for example, an injection of constant hyperpolarizing current (Aizenman and Linden, 1999). Thus, adaptation could be seen as change in neurons activity toward a typical or expected (predicted) level $\left(\tilde{x}\right)$.

To investigate effect of adaptation on neuronal processing, we implemented a brain-inspired adaptation mechanism in the units in our network. For this, during the clamped phase from time step 8, the activity of each neuron was nudged toward the predicted state (Figure 4B). Specifically, the activity of neuron *j* at time step *t* was calculated as: $x_{j,t}=a*\tilde{x}+\left(1-a\right)*\sum_{i}\left(w_{i,j}*x_{i,t-1}\right)$, where 0 ≤ *a* ≤ 1 is a parameter denoting strength of adaptation. For example, for *a=0*, the adaptation is equal to zero, and the network activity is the same as in original network described in Figure 2. To update synaptic weights, we used the same learning rule as in Figure 1: $\Delta \,w_{i,j}=x_{i}\,\left(x_{j}-{\tilde{x}}_{j}\right)$, but here *x*_*j*_ represents clamped activity with added adaptation, which can also be denoted as *x*_*A*_. Interestingly, networks with implemented adaptation achieved better accuracy than the same networks without adaptation (Supplementary Information). This could be due to the fact that if an activity in the clamped phase is much different from an expected activity without the clamp, then learning may deteriorate as those two network states could be in different modes of the energy function (Scellier and Bengio, 2017). Adaptation may reduce this problem by bringing clamped state closer to expected. To give an analogy, if part of a car is occluded by a tree, then, purely by sensory information, we cannot say what is behind that tree. However, based on our internal model of the world, we know what shape a car is, and, thus, we can assume that the rest of the car is likely behind the tree. Similarly, neuronal adaptation may allow a neuron to integrate the input information with predictions from its internal model, and then adjust its activity based on this combined information leading to a more appropriate response.

## Hypothesis and Theory

### Predictive Adaptation as a Signature of Consciousness

It is largely accepted that consciousness is a gradual phenomenon (Francken et al., 2021). It was also suggested that even a single cell may have a minimum level of consciousness, based on the complexity of behavior and complexity of information-processing within each cell (Reber, 2016; Baluška and Reber, 2019). For example, every single cell contains large biochemical networks, which were shown to make decisions and to perform computations comparable to electrical logic circuits (Supplementary Figure 2; McAdams and Shapiro, 1995). This allows for highly adaptive behavior, including sensing and navigating toward food, avoiding a variety of hazards, and coping with varying environments (Kaiser, 2013; Boisseau et al., 2016). For instance, single-celled organisms were shown to be able to “solve” mazes (Tero et al., 2010), to “memorize” the geometry of its swimming area (Kunita et al., 2016), and to learn to ignore irritating stimulus if the cell’s response to it was ineffective (Tang and Marshall, 2018). Moreover, single-celled microorganisms were shown to predict environmental changes, and to appropriately adapt their behavior in advance (Tagkopoulos et al., 2008; Mitchell et al., 2009). Those complex adaptive behaviors were proposed to resemble cognitive behavior in more complex animals (Lyon, 2015). This likely requires organism to build some sort of internal predictive model of their own place in the environment, which could be considered as a basic requirement for consciousness.

The results presented in Figure 3 suggest that the level of consciousness could be related to the amount of surprise. This is also supported by results from human EEG studies, where the neuronal signature of surprise: P300, closely reflects conscious perception (Del Cul et al., 2007; Dehaene and Changeux, 2011). Here, we propose that in a neuron, adaptation could be seen similarly to P300, as a measure of surprise, and thus, it could provide an estimate of the level of “conscious cellular perception.” Specifically, as described above, surprise could be defined as a difference between actual (*x*) and predicted activity ($\tilde{x}$). Because adaptation changes neurons’ activity toward a predicted activity level, thus, the size of adaptation (|*x*−*x*_*A*_|) is directly related to the size of surprise: (|$x-\tilde{x}$|). Therefore, we propose to define the single-neuron correlate of consciousness (sNCC) as the magnitude of neuronal adaptation sNCC = |*x*−*x*_*A*_|, (Figure 4B). Based on this, we hypothesize that single-cell predictive adaptation is a minimal and sufficient mechanism for conscious experience.

### Generalized Definition of Consciousness as a Process of Surprise Minimization

**FIGURE 5:**
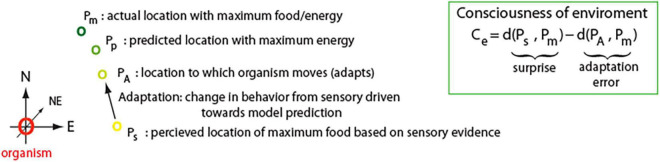
Insert: Consciousness (C_*e*_) is defined here as a surprise: distance d(,) between obtained sensory information (P_*s*_) and expected information. However, if system cannot appropriately adapt based on that information, then conscious perception is reduced (adaptation error). Thus, Consciousness is a function of surprise and ability of organism to adapt to minimize that surprise. Expected information is denoted by P_*p*_ and it is calculated by internal predictive model, which based on partially available data tries to approximate actual state of the environment (P_*m*_). Schematic on the left illustrates concept of C_*e*_ for sample organism living in 2D environment (see main text for details).

First, we will explain the main ideas using a simplified example, then later, we will present how it can be generalized. Let us have a two-dimensional environment, where at each location P, there is a certain amount of food. There is also an organism that wants to go to a location with the highest amount of food. That organism does not know exactly how much food there is at any given location, but based on past experience, the organism has an internal model of the environment to help with predictions. For instance, let us assume that the maximum concentration of food *(m)* is at point P_*m*_, but the smell of food comes from the direction of point: P*_*s*_*, where *s* stands for sensory evidence (Figure 5). However, the concentration of food in the past was highest in the North direction. The internal model combines this information and predicts the highest probability of food in the North East direction at point P*_*p*_*, where *p* stands for predicted. Based on this, the organism adapts and moves toward P*_*p*_* to location P_*A*_, where _*A*_ stands for adaptation. When the organism arrives to P_*A*_ location, then it can compare the actual amount of food at that location with the predicted one, and update the internal model accordingly. Thus, by combining sensory information and internal model predictions, our organism was able to adapt its behavior more appropriately.

In the above-described case, we could say that our organism was quite conscious of its environment, as it made close to optimal decision. We can quantify it by measuring how close to optimal location an organism moved: d(P_*A*_, P_*m*_), as compared if it would move in reflex-like fashion to location that is purely determined by sensory stimulus: d(P_*s*_, P_*m*_), where d(.,.) denotes a distance between 2 points. Specifically, we can define organism consciousness of environment as C_e_ = d (P_s_, P_m_) – d (P_A_, P_m_), (Figure 5, insert). It is worth noting that if an organism has a good model of external environment to correctly predict location with maximum food, then: P_p_ ≈ P_m_, and thus, the first term in C_e_: d(P_s_, P_m_) ≈ d(P_s_, P_p_), where this distance d (P_*s*_, P_*p*_) between sensory evidence (P_*s*_) and model prediction (P_*p*_) is a description of surprise. The second term in C_e_: – d (P_A_, P_m_), describes how far organism is from location with maximum food/energy (P_*m*_) after it made adaptation (P_*A*_). This could be seen as an error term, which could arise if predictive model is incorrect or if organism is unable to move exactly to the predicted location. Hence, according to the above definition of C_*e*_, consciousness is equivalent to surprise, if error term is 0, which would be the case for an organism to able to perfectly adapt.

Although, we used here a two-dimensional environment as an example, this can be generalized to a high-dimensional sensory space. Let us consider a simple organism which can sense concentration of 10 substances in a deep ocean. As organism swims, it changes its position in 3D space, but more importantly, concentration of 10 substances indicating location of food and predators also changed with each movement. Thus, 3D space translates to 10D sensory space, which is more relevant to that organism behavior. Therefore, distances d in C_*e*_ = d (P_*s*_, P_*m*_) − d (P_*A*_, P_*m*_), may be more appropriately calculated in sensory space of that organism, instead of the standard 3D spatial coordinates. For example, we implicitly used idea of sensory space in case of neurons shown in Figures 3, 4. Neuron senses its local environment through variety of channels located especially in synapses. Activity of a neuron affects other neurons, which through feedback loops change synaptic inputs to that neuron, and thus, its sensory environment. Because neuron gets energy from blood vessels, which dilation is controlled by coordinated activity of local neurons, therefore, neuron may “want” to move in the sensory space corresponding to activity patterns resulting in the most local blood flow. Therefore, change in neuron activity is equivalent to a movement in a chemical sensory space, where different locations in that space correspond to different amount of energy obtained by a neuron. For that, the word “environment” in C_*e*_ refers to this highly dimensional sensory space rather than that of the typical 3D space.

This generalization to sensory space also allows to see notions introduced earlier in Figures 3, 4 as special cases of environmental consciousness C_*e*_. For example, when organism has the perfect model of external environment, then it can correctly predict the location with maximum food, thus, P_*p*_ = P_*m*_, as we have explained before. However, if that organism can also move exactly to predicted location such that: P_*A*_ = P_*p*_, then, also P_*A*_ = P_*m*_. In such case, an adaptation error d(P_*A*_, P_*m*_) becomes 0, and thus, C_*e*_ = d (P_*s*_, P_*m*_). Considering the above case that P_*m*_ = P_*A*_, C_*e*_ can also be expressed as C_*e*_ = d (P_*s*_, P_*A*_), which is a distance by how much an organism moved or adapted. Thus, in case of the neuron described in Figure 4, C_e_=d (P_s_,P_A_) ≈ d |*x*, *x*_*A*_| = |*x* − *x*_*A*_| = sNCC. Similarly, as mentioned earlier, C_*e*_ becomes equivalent to surprise if organism perfectly adapts (P_*A*_ = P_*p*_ = P_*m*_). In such case, adaptation error is zero, and we can write ${\text{C}}_{\text{e}}=\text{d}\,\left({\text{P}}_{\text{s}},P_{\text{p}}\right)\approx \left|x-\tilde{x}\right|$, which is the distance between the stimulus-evoked activity and the model prediction, which we used to quantify the skill consciousness in Figure 3. Thus, C_*e*_ is a function of surprise and ability of organism to adapt to minimize that surprise.

Note that surprise and adaptation could be considered as contributing to C_*e*_ on different timescales, with synaptic changes gradually minimizing surprise over a long period of time, and with neuronal adaptation changing neuronal firing rapidly within 10–100 ms. When an organism is learning a new skill, then activity driven by bottom-up signals is different from activity provided by top-down teaching signals, which results in a higher surprise term. However, if neurons cannot adapt their activity accordingly (e.g., when biochemical processes mediating adaptation within a neuron are blocked), then adaptation error will be as large as the surprise term, resulting in C_*e*_ = 0 and, thus, in no conscious experience. Therefore, the surprise term could be interpreted as “potential consciousness,” meaning the maximum possible consciousness to a given stimulus. Synaptic strength gradually changes over a period of learning, resulting in slow changes in “potential consciousness.” However, when a stimulus is presented, and neurons rapidly adapt their activity toward the predicted level, it reduces the adaptation error term and results in C_*e*_ > 0, and, thus, in conscious perception within a fraction of a second.

### Hypothesis Validation

A hypothesis, by definition, should generate testable predictions. Our main hypothesis is that the *neuronal adaptation is a neuronal correlate of consciousness*. This implies that neurons and, thus, brains, without adaptation cannot be conscious. Therefore, our hypothesis predicts that any mechanism which affects neuronal adaptation will also affect consciousness. This prediction was shown to be correct for a diverse group of neurochemicals involved in sleep and anesthesia, which also alter the neuronal adaptation. For instance, levels of multiple neuromodulators in the brain such as serotonin, noradrenaline, and acetylcholine are significantly different between waking and sleeping in REM or non-REM stages (España and Scammell, 2011). Whole-cell voltage-clamp recordings *in vitro* in the pyramidal neurons have demonstrated that all those neuromodulators also affect neuronal adaptation (Satake et al., 2008). Similar results were obtained when testing various substances used for anesthesia, such as urethane (Sceniak and MacIver, 2006), pentobarbital (Wehr and Zador, 2005), and ketamine (Rennaker et al., 2007). Moreover, it was shown that a large variety of anesthetics, including butanol, ethanol, ketamine, lidocaine, and methohexital are blocking calcium-activated potassium channels, which mediate neuronal adaptation (Dreixler et al., 2000). Interestingly, considering a broad spectrum of molecular and cellular mechanisms affected by different anesthetic compounds, there remains significant uncertainty of what is the single mechanism underlying anesthesia (Armstrong et al., 2018). Our theory suggests that what all anesthetics could have in common is the ability to disturb neuronal adaptation. Thus, our theory clearly provides testable predictions, which could either be invalidated or validated by using pharmacological and electrophysiological methods (see also “Limitation” section for more discussion on this topic).

### Predictive Adaptation as a Step Toward a Unified Theory of Consciousness

Important consequence of a neuron adapting its activity toward a predicted level is that it allows neurons to exchange information about its predictions. Thus, neuron output activity is not exclusively driven by its synaptic inputs, but it is also a function of its internal predictive model. Below, we will briefly describe a few of the most prominent studies, as well as the theories of consciousness [for in-depth reviews see Francken et al. (2021) and Seth (2021)]. We will particularly focus on outlining the differences and similarities to our theory of predictive adaptation, and how it may provide a theoretical basis for connecting diverse theories of consciousness.

#### Connection to Optical Illusions

Exchanging predictions among neurons may explain multiple phenomena linked to conscious perception, such as optical illusions. For example, let us consider a neuron tuned to detect horizontal lines. Such neuron may learn that even when feed-forward inputs are not exactly consistent with a line (e.g., due to partial occlusion), then later on, it usually receives a top-down signal indicating detection of a line due to combining information from other parts of the image by higher cortical areas. Thus, in the case of an image with illusory contours, this neuron may receive less activation from feed-forward inputs, as parts of the lines are missing. However, based on experience with occluded objects, that neuron may predict that it will soon receive top-down signals indicating a line, thus, in expectation it will increase its activity toward predicted levels. Consequently, other neurons receiving this predictive information are more likely to interpret it as a line, resulting in positive feedback loops and coherent perception of a line.

Similar explanation could also be applied in case of ambiguous images like the Rubin vase (face) optical illusion. If a set of neurons in the association cortex receives inputs suggesting an image of a face, then they will increase their activity accordingly toward that “believe,” triggering a global activity pattern giving a single perception of a face.

#### Connection to Global Neuronal Workspace Theory

As described above, a large-scale neuronal convergence to a single “believe” is very similar to a theory of global neuronal workspace (GNW) (Baars, 2002; Dehaene and Changeux, 2011). Briefly, GNW states that an organism is conscious of something, only when many different parts of the brain have access to that information. Additionally, if that information is contained only in the specific sensory or motor area, then the organism is unconscious of that something. In our theory, consciousness is on a continuous scale. However, if an activity is different from what is expected across the many parts of the brain, then our measure of C_*e*_ will also be larger as compared to a single brain area, and because the brain is a highly non-linear system, C_*e*_ could be orders of magnitude larger when the difference between expected and predicted signal is exchanged in feedback loops across the entire brain. Thus, if the brain during waking has close to maximum C_*e*_, and low C_*e*_ during, for example, sleep, with intermediate values of C_*e*_ existing shortly during transition between those states, then this could reconcile the apparent difference between both theories. It is worth noting also that according to the GNW theory, a key signature of information accessing consciousness is the P300 component, which as mentioned earlier reflects surprise (Donchin, 1981; Mars et al., 2008). This is similar to our theory where C_*e*_ is defined in terms of surprise (Figure 5). Therefore, taken all together, GNW may be seen as a special case in our theory, where C_*e*_ is discretized to have only two values.

#### Connection to Integrated Information Theory

Our theory is also consistent with the main ideas of integrated information theory (IIT). The IIT quantifies consciousness as the amount of information generated by an integrated complex of elements above and beyond the information generated by its individual parts, which is denoted as Phi (Tononi, 2015; Tononi and Koch, 2015). Similarly, in our case, if two cells can communicate, then this will allow each of them to make better predictions and, thus, to increase combined C_*e*_, by reducing error term: d (P_*A*_, Pm). For instance, if cell #1, just by chance, has more receptors to detect substance *s1*, and cell #2 has slightly more receptors for substance *s2*, then by communicating predictions to each other, both cells will be able to better detect food, which secretes *s1* and *s2* [i.e., the wisdom of crowds (Friedman et al., 2001)]. This simplified example can be directly extended to neurons, where each has unique pattern of connections, thus, partly providing novel information to other neurons. However, there is one important difference between Phi and C_*e*_. While Phi can be computed based purely on connectivity pattern, C_*e*_ also depends on stimulus. If stimulus is unexpected, then surprise term d (P_*s*_, Pm) will increase, and thus, even without any change in network architecture, organism will be more conscious of that stimulus. However, on average, elements with more complex connectivity patterns, which have higher integrated information Phi, will also have higher C_*e*_, as more information sources will be available for each element to improve predictions, thus, reducing error term in C_*e*_.

#### Connection to Attention Schema Theory

It was also proposed that consciousness requires building an internal model of incoming information. For example, the brain constructs a simplified model of the body to help monitor and control movements, and similarly, at more abstract level, it may construct an internal model of attention, which could form a basis for consciousness (Graziano and Kastner, 2011; Graziano and Webb, 2015). In our theory, an internal model is a crucial part of defining the consciousness. Although our predictive model is at the single-cell level, communication between neurons could allow to form more complex models at the network level. Note that due to neuronal adaptation toward predicted activity, each neuron sends information to others, reflecting its internal model predictions. Thus, neurons in higher areas build their internal models based on combining information from other neuron models. This suggests that higher-order models, like the model of attention proposed by Graziano, could be a direct consequence of building the brain from elements with internal models as described by our theory.

#### Connection to Predictive Processing

Our theory is closely related to the predictive processing framework. This theoretical framework posits that the brain’s overall function is to minimize the long-term average prediction error (Hohwy and Seth, 2020). It also proposes that to accomplish this process, the brain needs to have a generative model of its internal and external environment, and continually update this model based on prediction error (Friston, 2005, 2010; Friston et al., 2017). The precursor of the predictive processing idea could be traced back to a 19th century scholar named Hermann von Helmholtz (Von Helmholtz, 1867). He suggested that the brain fills in some missing information to make a better sense of its surrounding environment. As in the earlier example of a car behind a tree, the brain fills in the occluded parts to provide the most likely picture of the surrounding world. Over the recent years, predictive processing has gained significant experimental support [see for review Walsh et al. (2020)]. There were also proposed predictive computational models of vision, illustrating how top-down processing can enhance bottom-up information (McClelland and Rumelhart, 1981; Rao and Ballard, 2005). An important theoretical advancement was made, when it was shown that predictive processing can be understood as Bayesian inference to infer the causes of sensory signals (Friston, 2003, 2005). This provided a mathematically precise characterization of the predictive processing framework, which was further generalized in the form of the free energy principle (Friston, 2010). Our theory is fully consistent with this framework. However, our work provides three novel and important contributions to predictive processing:

(1) We derived mathematically that the predictive processing maximizes metabolic energy of a neuron (Figure 1), which provides biologically bound theoretical basis for predictive processing framework.
(2) Based on the above theoretical considerations and based on computational simulations, we showed that a single neuron could be the basic element for building diverse predictive networks (Figures 2, 3). This offers a solution to how predictive processing could be implemented in the brain without the need for precisely designed neuronal circuits or special “error units.”
(3) Most importantly, we showed that predictive neuronal adaptation could be the mechanism for conscious processing (Figure 4) and based on this, we proposed a quantitative definition of consciousness (Figure 5).

## Limitations

While the present study offers a novel theoretical model of consciousness derived from basic principles of maximizing metabolic energy, this also comes with caveats that should be considered. In the absence of a generally accepted definition and measure of consciousness, all theories of consciousness, including ours, are unfortunately more speculative than typical theories in mostly other areas of science. For instance, to date, no theory has convincingly demonstrated yet how neuronal mechanisms can generate a specific conscious experience. Similarly, with our theory, it has yet to be shown that connecting billions of adaptive neurons could result in subjective feelings of “self,” which is typically considered as consciousness. Here, as a step toward addressing this problem, we described how single-neuron-level predictive processes could be related to consciousness of skills at the organism level (Figure 3). However, the caveat here is that “skills consciousness” (as well as “consciousness”) does not have a well-defined measure, thus, changes in skill consciousness during learning are only described in loose qualitative terms. This needs to be more rigorously measured in the future to allow for more quantitative comparison to our model.

The related problem in theories of consciousness is the difficulty in proving causal mechanisms of consciousness. For example, in our definition of consciousness, the first term represents “surprise” (Figure 5), and as we described earlier, there is strong a experimental evidence relating surprise (e.g., P300) to conscious perception in humans. However, the caveat is that it is also possible that surprise could be correlated with consciousness without causing it, thus, experiencing surprise and acting on it may not be sufficient to create consciousness. Similarly, we described experimental evidence showing that a diverse group of neurochemicals involved in sleep and anesthesia also affects neuronal adaptation. However, this is also only a correlation, and to prove that neuronal adaptation causes consciousness, experiments controlling multiple confounding factors, and selective blockage of adaptation would be needed to provide a more conclusive answer.

One interesting feature of our definition of consciousness is its simplicity and scalability: the same simple equation can describe consciousness at the single-cell level as well as at the whole organism level. However, this could be taken as an argument against our theory, as the claim of consciousness in the single cell or in a robot could be considered as a “far cry” from the typically understood notion of consciousness. This is a valid objection. To address this semantic problem, we introduced a broader term, “consciousness of environment” (C_*e*_; Figure 5). What we are proposing in this manuscript is that the consciousness of environment is on a continuous scale, and the consciousness that we are experiencing as humans is just an extreme case of the same process. To give an analogy, the celestial movement of planets was considered to be governed by different laws than earthly objects, but now we understand that the same gravitational laws could be used to describe the movement of objects at both scales, which we suggest could be similar with consciousness. Unfortunately, we are still missing experimental means to precisely measure consciousness, which makes theories of consciousness more difficult to verify, and thus, more speculative.

Moreover, surprise minimalization could also be achieved by other means than the intracellular predictive mechanism proposed here. For instance, multiple predictive coding networks have been developed, with specially designed neuronal circuits including “error units,” which allow for comparing expected and actual activity (Rao and Ballard, 2005; Bastos et al., 2012; Whittington and Bogacz, 2017; Sacramento et al., 2018). Such networks can be trained using other biological learning rules, like spike-time-depended plasticity [STDP; (Bi and Poo, 2001)] or some variation of Hebbian learning [e.g., BCM (Bienenstock et al., 1982)]. Thus, it is possible that consciousness in neuronal system may be created by predictive mechanisms implemented only at the network level. One problem with predictive coding only at the circuit level is that it requires precise connectivity, which could be difficult to achieve, considering the complexity and variability of neuronal dendritic trees. Here, deriving from the basic principle of metabolic energy maximization, we suggest that predictive neurons could provide an elementary unit from which a variety of predictive circuits could be built, thus solving the above implementation problem. Therefore, in addition to intracellular predictions, neurons may form predictive circuits, giving rise to enhanced predictive abilities that increase the level of consciousness in an animal, as discussed above in relation to attention schema theory. Those network-level interactions may lead to a rapid and exponential-like increase in C_*e*_. However, contrary to many other theories of consciousness, we suggest that this increase in Ce will not result in qualitative change, and that consciousness from single-celled organisms to humans could be described on a continuous scale, as the same adaptive process of surprise minimization.

## Data Availability Statement

The datasets and our code is freely available online: https://people.uleth.ca/∼luczak/PredC/. The code to reproduce results of this study is also provided in Supplementary Material.

## Author Contributions

AL conceived the project, analyzed data, performed computer simulations, and wrote the manuscript. YK performed computer simulations and contributed to writing the manuscript. All authors contributed to the article and approved the submitted version.

## Conflict of Interest

The authors declare that the research was conducted in the absence of any commercial or financial relationships that could be construed as a potential conflict of interest.

## Publisher’s Note

All claims expressed in this article are solely those of the authors and do not necessarily represent those of their affiliated organizations, or those of the publisher, the editors and the reviewers. Any product that may be evaluated in this article, or claim that may be made by its manufacturer, is not guaranteed or endorsed by the publisher.
