# Supplementary material for: Predictive Neuronal Adaptation as a Basis for Consciousness
Source: Front Syst Neurosci. 2022 Jan 11;15:767461. doi: 10.3389/fnsys.2021.767461 (PMC8789243; doi:10.3389/fnsys.2021.767461)
Supplement: Supplementary file 1 [file Data_Sheet_1.pdf]

**Supplemental Materials**

Artur Luczak & Yoshimasa Kubo. Predictive neuronal adaptation as a basis for consciousness. *Frontiers in Systems Neurosci*. 2021.

***Derivation of predictive learning rule.***

Intuitively, it makes sense that planning, i.e. making educated predictions, can improve the success of organisms in accessing more energy resources. Making good predictions can also allow to better solve complex tasks (Chalmers et al., 2017). Below we outline the main steps of the derivation, which shows that for a single neuron, maximizing metabolic energy is best achieved by predicting future activity [for details see (Luczak et al., 2022)]. Energy supplied to neuron $j$ ($E_b$) comes from local blood vessels controlled by the combined activity of local neurons, which can be described as: $E_b = b_2(\sum_k x_{k,t+n})^{\beta_2}$ , where $x_k$ represents spiking activity of neuron $k$ from a local population of $K$ neurons ($k \in \{1, \ldots, j, \ldots K\}$), $t$ represents current time, $n$ is a small time increment, $\beta_2$ describes a non-linear relation between activity and energy (Devor et al., 2003), and $b_2$ is a proportionality constant. Similarly, energy used on electrical activity can be written as a power function of the sum of its synaptic inputs: $E_{ele} = b_1(\sum_i w_{ij} x_{i,t+n})^{\beta_1}$ (Devor et al., 2003; Luczak et al., 2022). Note that in this derivation $x$ does not refer specifically to the clamped phase but rather to neuron activity in general (we will make a connection to clamped activity at the end of this section). Cellular housekeeping cost $E_h$ is here considered to be a constant. Therefore, the equation for energy balance for a neuron $j$ can be written as:

(Eq. S1): $E_j = E_b - E_{ele} - E_h = b_2(\sum_k x_{k,t+n})^{\beta_2} - b_1(\sum_i w_{ij} x_{i,t+n})^{\beta_1} - E_h$

In simulations and in experimental data we showed that for small $n$, the activity of neuron $j$ at time $t+n$ could be approximated by a linear function of its activity at earlier time step $t$, such as: $x_{j,t+n} = \lambda_j x_{j,t}$, where $\lambda$ is a regression coefficient (Luczak et al., 2022). Thus Eq. S1 can be rewritten as

(Eq. S2): $E_j = b_2(\sum_{k \neq j} x_{k,t+n} + \lambda_j x_{j,t})^{\beta_2} - b_1(\lambda_j \sum_i w_{ij} x_{i,t})^{\beta_1} - E_h$

Using the gradient ascent method, we can calculate the change in weights to maximize energy balance:

(Eq. S3): $$\Delta w_{ij} = \frac{\partial E_j}{\partial w_{ij}} = x_i \lambda_j \beta_2 b_2(\textstyle\sum_{k \neq j} x_{k,t+n} + \lambda_j \sum_i w_{ij} x_{i,t})^{\beta_2 - 1}$$
$$- x_{i,t} \lambda_j \beta_1 b_1(\lambda_j \sum_i w_{ij} x_{i,t})^{\beta_1 - 1}$$

Note that in Eq. S3: $\lambda_j \sum_i w_{ij} x_{i,t} = \lambda_j x_{j,t} \approx \tilde{x}_j$ , thus this term corresponds to predicted future activity: $\tilde{x}_j$. We will also denote a population activity $\sum_{k \neq j} x_{k,t+n}$ as: $\hat{\tilde{x}}$ , which simplifies Eq. S3 to:

(Eq. S4): $\Delta w_{ij} = x_i \lambda_j \beta_2 b_2 (\hat{\tilde{x}} + \tilde{x}_j)^{\beta_2 - 1} - x_{i,t} \lambda_j \beta_1 b_1 \tilde{x}_j^{\beta_1 - 1}$

Considering experimental results from (Devor et al., 2003) and computational results from (Luczak et al., 2022), we can use $\beta_1 = 2$ and $\beta_2 = 2$, which allows to simplify Eq. S4 to:

(Eq. S5): $\Delta w_{ij} = \alpha_3 \, x_{i,t} \left( \alpha_4 \hat{\tilde{x}} - \tilde{x}_j \right)$ , where $\alpha_3 = 2\lambda_j ( b_1 - b_2)$, and $\alpha_4 = \frac{b_2}{( b_1 - b_2)}$

In the equation above, $\alpha_3$ can be denoted as simply a learning rate $\alpha$, and for notation simplicity, index $t$ in $x_{i,t}$ can be omitted as it refers to current time step. Moreover, local population activity $\hat{\tilde{x}}$ provides synaptic inputs to neuron $j$, thus $x_j \approx \sum_k w_{k,j} x_k \approx \alpha_4 \sum_k x_k \approx \alpha_4 \hat{\tilde{x}}$. This suggests that the activity of a single neuron can be approximated from local population activity, which is supported by experimental evidence (Tsodyks et al., 1999; Harris et al., 2003; Luczak et al., 2004; Luczak et al., 2009). Thus, equation for modifying synaptic weights could be expressed as:

(Eq. S6): $\Delta w_{ij} \approx \alpha x_i \, (x_j - \tilde{x}_j)$

This shows that maximizing future energy balance requires a neuron to predict its future activity ($\tilde{x}_j$). However, it should also be noted that although this analytical derivation of the synaptic learning rule provides a novel step to link predictive learning models to metabolic activity, it requires to greatly simplify the description of metabolic processes to only a few most important variables. The predictive learning rule presented here was validated in computer simulations (Fig. 3 and (Luczak et al., 2022)), but the biological accuracy of this simplified model and assumptions used in this derivation need further investigation.

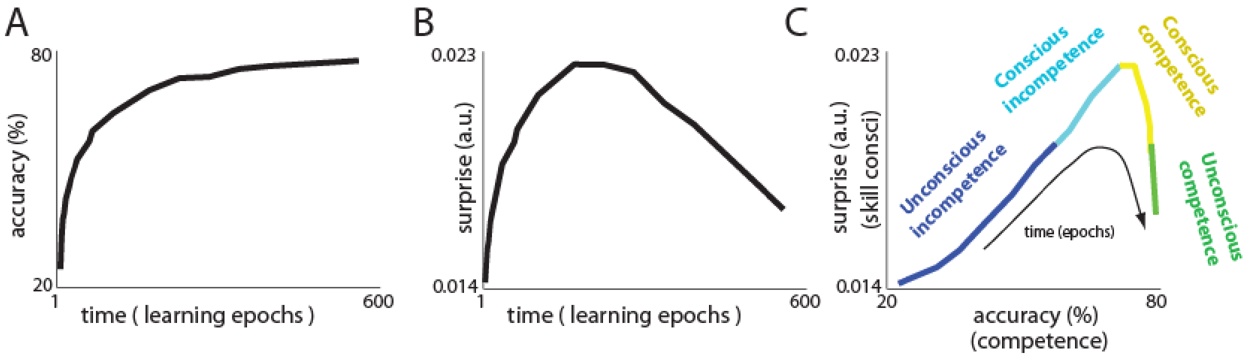


**Suppl. Figure 1**. Neuronal surprise for a convolutional network trained with predictive learning rule on CIFAR-10 dataset. Plot convention is the same as in Fig. 3 in the main text. (A) Learning curve. (B) Change in neuronal surprise averaged over all neurons. (C) Neuronal surprise (proxy of skill consci.) vs accuracy (a.k.a. competence). Those results show that a convolutional network with predictive learning rule also shows behavior similar to stages of conscious competence.

The details of this convolutional network were described in (Luczak et al., 2022), and code is available at: https://github.com/ykubo82/bioCHL/tree/master/conv . Shortly: the network had an input layer of size: 32x32x3, corresponding to the size of a single image with 3 color channels in CIFAR-10 dataset (this dataset consists of 5000 training and 1000 test images for each of 10 classes (Krizhevsky and Hinton, 2009)). The network had two convolutional and pooling layers followed by one fully connected output layer. The filter size for all the convolutional layers is 3x3 with stride 1, and the number of filters is 256 and 512 for the first and second convolutional layers, respectively. This network with predictive learning rule achieved test accuracy of 79.97% (chance level: 10%), which is comparable accuracy to training the same network with backpropagation through time algorithm (79.12%) (Luczak et al., 2022).

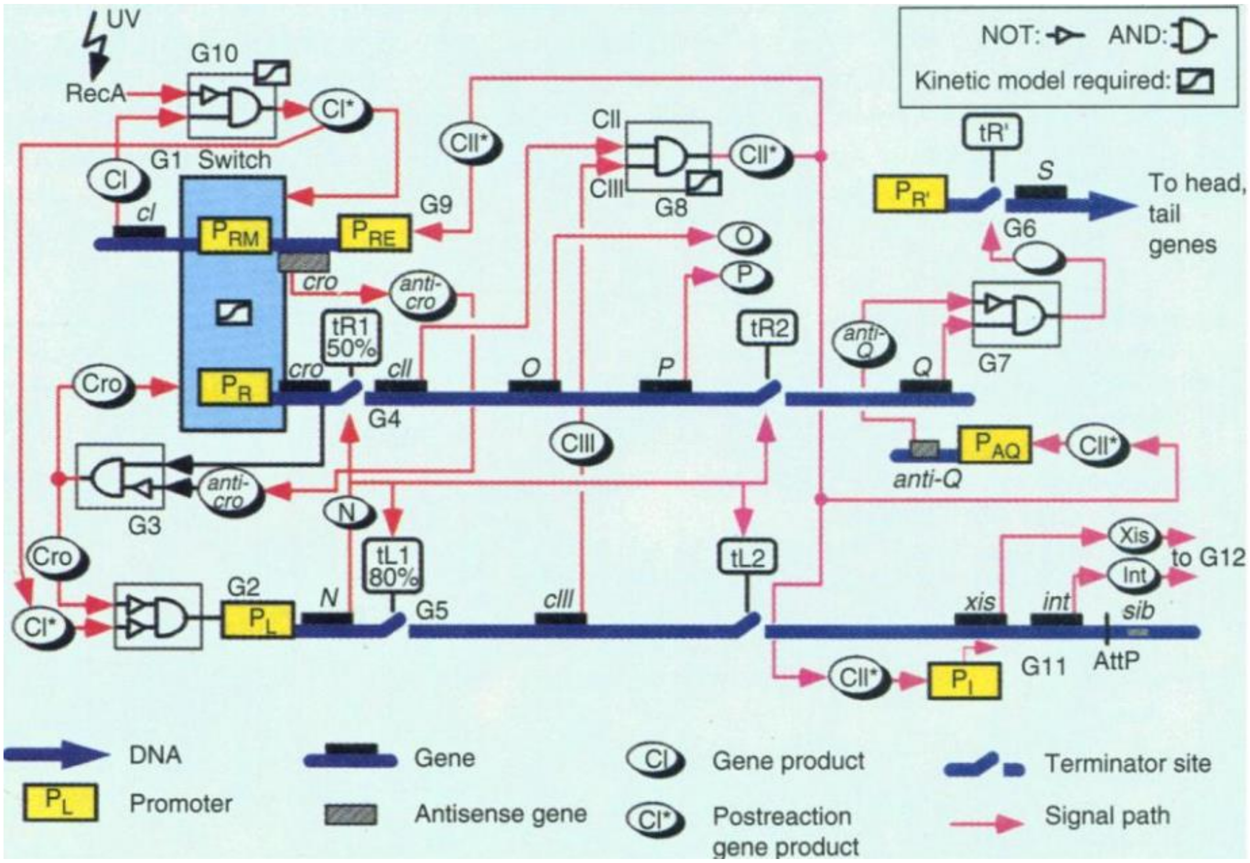


**Suppl. Figure 2**. Sample biochemical circuit determining decision in a phage (McAdams and Shapiro, 1995). This illustrates that biochemical interactions can perform complex computations comparable to electrical logic circuits.

***Sample code for network with predictive learning rule and with neuronal adaptation.***

This code demonstrates how surprise is calculated to produce results presented in Fig. 3. To reduce simulation time, we present here a network with only 50 hidden units, and with the number of training epochs set to 300. We also reduced here the number of time steps for each stimulus presentation from 120 to 20, as compared to our previous work (Luczak et al., 2022). The predicted activity ($\tilde{x}$) was calculated only from the first 5 time steps, and the clamped teaching signal was switched on at time step 8 (see Fig. 1B for illustration). To calculate a neuron's predicted or expected activity, we used a linear function of its past activity: $\tilde{x}_{(t)} = \lambda_{(1)}^{*} x_{(t-1)},+\ldots+ \lambda_{(n)}^{*}\ x_{(t-n)}$ + *constant*, where $x_{(t-n)}$ is past activity at time step *t-n*, *n* is a maximum number of time steps contributing to the prediction, and $\lambda_{(n)}$ is a coefficient describing how past activity at time step *t-n* contributes to predicted activity at time *t*. We tested the validity of this linear formula in simulations and in experimental data (Luczak et al., 2022). Surprise is calculated as an absolute difference between clamped phase activity and predicted activity, and it is averaged over all neurons and all stimuli presented in a single training epoch (see supr_jj variable in code below). As maximum values of neuronal activity in our network are typically around 1, this sets the maximum limit for values of surprise. In Fig. 3 we presented results averaged over 100 simulations. In the code below, for purpose of demonstration we only run one simulation.

The effect of neuronal adaptation on improving network performance will be investigated in detail in future work. However, it can be exemplified using the code below. To do so, the number of training epochs should be increased to 5000 (itr variable), and the value of adaptation set to 0.7 (adp variable). As adaptation reduces the difference between clamped and predicted activity, the learning rate can be increased to ~0.6 (ler_rt). For comparison, a network without adaptation (itr =0) could be best trained with learning rate: ler_rt = 0.015. To speed up computations we also set $\tilde{x} = x_F$ (do_pred = 0) for both comparisons. This could be interpreted as neurons doing perfect predictions, where predicted activity is set exactly to the value of free phase activity. This is justified by our previous results showing that correlation between $\tilde{x}$ and $x_F$ was R=1$\pm$0.0001 SD (Luczak et al., 2022). Using parameters described above we achieved a maximum accuracy of 96.3% for the network with adaptation, and 91.9% without adaptation on test dataset (cross-validation). Note that using networks with a relatively small number of neurons made the MNIST task more difficult for the networks to solve, thus making it easier to see differences in performance when using adaptation.

```
%%- Matlab code to reproduce results presented in Fig. 3
% - author: Artur Luczak @ University of Lethbridge
% - code is free to use and modify under MIT License
% - It calculates surprise and adjusts neuron weights using predictive learning rule
% - As input it uses MNIST dataset (LeCun et al. 1998) converted to .mat format
% - This code and .mat data is available at: https://people.uleth.ca/~luczak/PredC/

clear all; warning off

itr = 300; %-- number of learning epochs
ler_rt = 0.15; %-- learning rate
adp = 0.1; %-- strength of neuronal adaptation; if adp=0 then no adaptation. Must be <1
nr_exmp_cl = 400; %-  number of randomly selected examples for single training epoch (clamped phase)
nr_exmp = 2000;  %- number of examples for free phase ( must be >= nr_exmp_cl )
nr_exmp_all = 2000; %5000 %- number of images from each MNIST class to store in memory
dt = 0.8; %-- (1-dt) = influence of activity at previous step; if dt=1 then only current activity
sym_w = 1; %-- if 0 then no symmetric weights; otherwise set it to 1
recur = 0; %-- if == 1 then lateral connections within layer? (fully recurrent net)

do_pred = 1; %-- if 1 then use predicted activity
if do_pred == 0; nr_exmp = nr_exmp_cl; end
pr_st = 5; %-- number of time steps to use for predictions
%-- stim parameters
t_clmp = 20; %-- number of time steps for each stim presentation
t_dc = 8; %-- delay of teaching signal in relation to stim

img0 = zeros(28,28); img0( 5:24,6:26) =1; % figure; imagesc(img0)
f_img = find( img0(:) == 1 ); %-- find center region of img
dns = 1; %-down sampling factor
dat = zeros(nr_exmp_all*10,length(f_img(1:dns:end)));
clas = zeros(nr_exmp_all*10, 10);

for i=1:10
  eval(['load ''digit' num2str(i-1) '.mat'''])
  idxC = [1:nr_exmp_all]+nr_exmp_all*(i-1);
  dat( idxC,:) = D([1:nr_exmp_all]+0, f_img(1:dns:end))/256; %-- take only center region of img
  clas(idxC, i ) = 1;
end

netA = [ size(dat,2) 50 size(clas,2) ]; %- network architecture: size of [input, hidden layers, output layer]

nr_nr = sum( netA(2:end));
nr_inp = nr_nr + netA(1) + 1; %-- add number of input neurons + bias

w = randn( nr_nr, nr_inp )/100; %-- weights
w_msk = zeros(nr_nr, nr_inp);
n1=1;
```

```
for i=2:length( netA )-1 %-- loop for layers without lateral connections
  n2 = n1 + netA(i)-1;
  n3 = n1 + netA(i);
  n4 = n3+netA(i+1)-1;
  w_msk(n1:n2,n3:n4)=1; %- backward conn from next layer
  w_msk(n3:n4,n1:n2)=1; %- forward conn to next layer
  n1 = n1+netA(i);
end
if recur == 1;
  w_msk(1:nr_nr,1:nr_nr) = 1;%- connect all-to-all within layer
end
for i = 1:nr_nr; w_msk(i,i) = 0; end %-prevent self connections
w_msk(1:netA(2),nr_nr+1:nr_nr+netA(1)) = 1; %-- inputs only go to layer 1
for n1=1:nr_nr;  for n2=n1+1:nr_nr
  if sym_w == 1; w(n1,n2) = w(n2,n1); end %-- make weights symmetric
  w_msk(n1,n2) = w_msk(n1,n2); %-- feedback weights multiplied by gamma
end; end
w_msk(:,nr_inp) = 1; %-- each neuron get bias

w_All = zeros( itr,nr_nr,nr_inp);
r = zeros(nr_inp, nr_exmp, t_clmp ); %- array with activity during free phase
rp = zeros(nr_nr, nr_exmp_cl);
aa = zeros(nr_exmp_cl,nr_nr,nr_inp); aa1 = zeros(nr_exmp_cl,nr_nr,nr_inp);
train_acur = zeros(1,itr); supr_jj = zeros(1,itr);

for jj = 1:itr %- loop for epochs
  w0 = w;
  if sym_w == 1; for n1=1:nr_nr;  for n2=1:nr_nr
     w0(n1,n2) = (w(n2,n1) + w(n1,n2))/2; %-- make weights symmetric
  end; end; end
  w = w0;
  w = w .* w_msk;

  idx_rand = ceil( rand(1,nr_exmp)*size( clas,1)); %-- select random samples for training

  r(nr_nr+1:nr_nr+netA(1),:,1:t_clmp) = repmat( dat(idx_rand,:)',1,1, t_clmp); %-- add clamped inputs
  r(nr_inp, :, 1:end) = 1; %-- bias

  v = zeros(nr_exmp,nr_nr);
  v1 = zeros(nr_exmp_cl,nr_nr);

  for i = 1:t_clmp-1  %- calculate activity at each time step for free phase
    v = v*(1-dt) + squeeze(r(:,:,i)'*w')*dt;
    v( v<0 ) = 0;      %-- ReLu
    r(1:nr_nr,:,i+1) = v';
  end
```

```
a = r(:, 1:nr_exmp_cl, end ); %-- activity at convergence point for free phase

if do_pred == 1  %-- if 1 then predict sustained response from onset activity

  ex_tr = nr_exmp_cl+1:nr_exmp; %-- index of train examples for predictive model
  ex_pr = 1:nr_exmp_cl; %-- index for examples used for weights update

  act_tr = r(1:nr_nr,ex_tr, 2:pr_st );    %--for pred from only 1 neuron <<< start from step 2 <<<<
  act_pr = r(1:nr_nr,ex_pr, 2:pr_st );

  r_end = r( 1:nr_nr , ex_tr,end );
  for n = 1:nr_nr  %-- loop for each neuron to predict its future
    a0 = [ squeeze( act_tr( n ,:,:))'; ones(size(ex_tr))]' \ r_end( n ,:)'; %-- LS predictions
    rp(n,ex_pr) = [squeeze( act_pr( n ,:,:))'; ones(size(ex_pr))]' * a0;
  end
  rp( rp<0 ) = 0; %--ReLU
  ap = a;
  ap(1:nr_nr,:) = rp; %-- predicted activity at convergence point for free phase
  a = ap; %-- overwrite free phase with predicted
end %-- end do_pred

r1 = r(:,1:nr_exmp_cl,:); %- array with activity during clamped phase
r1(nr_nr-netA(end)+1:nr_nr,:, t_dc+1:t_clmp) = repmat(  clas(idx_rand(1:nr_exmp_cl),:)',1,1, t_clmp-
t_dc);  %- teaching signal to outputs

v1 = squeeze( r(:,1:nr_exmp_cl ,t_dc+1)'*w');
for i = t_dc+1:t_clmp-1  %- calculate activity at each time step for clamped phase
  v1 = v1*(1-dt) + squeeze(r1(:,:,i)'*w')*dt;
  v1 = v1*(1-adp) + a(1:nr_nr,:)'*adp; %-- adaptation toward final predicted activity
  v1( v1<0 ) = 0;      %-- ReLu
  r1(1:nr_nr-netA(end),:,i+1) = v1(:,1:nr_nr-netA(end))';  %
end

a1 = r1(:, :, end); %-- activity at convergence point for clamped phase
supr_jj(jj) = mean( mean( abs( a1(1:nr_nr,:) - a(1:nr_nr,:) ))); %-- average surprise

 for i = 1:nr_exmp_cl
   aa(i,:,:) = a(1:nr_nr,i)*a1(:,i)';%-- predicted activity * presynaptic activity
   aa1(i,:,:) = a1(1:nr_nr,i)*a1(:,i)';%-- clamped activity * presynaptic activity
 end

w_All( jj ,:,:) = w ;
dw = squeeze( mean( aa1 - aa, 1)*ler_rt ); %- calculate weights change
w = w + dw(1:nr_nr,:); %-- update weights

act0 = r(nr_nr-netA(end)+1:nr_nr,1:nr_exmp_cl, end );
[v_cls clas_p] = max( act0 );
```

```
  [v_cls1 clas1] = max( clas(idx_rand(1:nr_exmp_cl),:)' );
  train_acur(jj) = (1 - sum(clas_p ~= clas1)/length(clas1))*100;   %-- accuracy on train set

  if mod(jj,30) == 1
    disp(['epoch: ' num2str(jj) '; train accuracy: ' num2str(train_acur(jj)) '%'])
  end
end %-- jj

figure;plot( 1:itr, supr_jj ,'.');title('surprise')
figure;plot( 1:itr, train_acur ,'.');title('accuracy')
  p = polyfit(1:itr, train_acur,7); % polynomial fit
  acur_fit = polyval(p,1:itr);
  hold on; plot(  1:itr ,acur_fit,'k');
figure;plot( train_acur ,supr_jj,'.');title('accuracy vs surprise')
  p = polyfit(train_acur,supr_jj,7); % polynomial fit
  supr_fit = polyval(p,sort(train_acur));
  hold on; plot(  sort(train_acur) ,supr_fit,'k');



%% -- code to calculate accuracy on examples from test data set (cross-validation)
return %- comment out this line to run this code

nr_exmp2 = 100; %- number of test examples per class
dat2 = zeros(nr_exmp2*10,length(f_img(1:dns:end)));
clas2 = zeros(nr_exmp2*10, 1);
for i=1:10
  eval(['load ''digit' num2str(i-1) '.mat'''])
  idxC = [1:nr_exmp2]+nr_exmp2*(i-1);
  dat2( idxC,:) = D([1:nr_exmp2]+nr_exmp_all, f_img(1:dns:end))/256; %-- take only center region of img
  clas2(idxC ) = i;
end

r0 = zeros(nr_inp, nr_exmp2*10, t_clmp ); %- array with activity during free phase
test_acur=[];
for iw = 1:size( w_All,1) %- loop for epochs
  w = squeeze(w_All(iw,:,:));
  w0 = w;
  if sym_w == 1; for n1=1:nr_nr;  for n2=1:nr_nr
     w0(n1,n2) = (w(n2,n1) + w(n1,n2))/2; %-- make weights symmetric
  end; end; end
  w = w0;
  w = w .* w_msk;
  r0(nr_nr+1:nr_nr+netA(1),:,1:t_clmp) = repmat( dat2',1,1, t_clmp); %-- add clamped inputs at the end
  r0(nr_inp, :, 1:end) = 1; %-- bias

  v2 = zeros( nr_exmp2*10, nr_nr );
```

```
 for i = 1:t_clmp-1  %- calculate activity at each time step for free phase
   v2 = v2*(1-dt) + squeeze(r0(:,:,i)'*w')*dt;
   v2( v2<0 ) = 0;      %-- ReLu
   r0(1:nr_nr,:,i+1) = v2';
 end

 act0 = r0(nr_nr-netA(end)+1:nr_nr,:, end );
 [v_cls clas_p] = max( act0 );
 test_acur(iw) = (1 - sum(clas_p' ~= clas2)/length(clas2))*100;%-- accuracy on test set
end
figure;plot( test_acur,'.'); grid on; title('test accuracy')
disp(['maximum test accuracy: ' num2str(max(test_acur))])
```

## Supplemental References


Chalmers E, Contreras EB, Robertson B, Luczak A, Gruber A (2017) Learning to predict consequences as a method of knowledge transfer in reinforcement learning. IEEE transactions on neural networks and learning systems 29:2259-2270.

Devor A, Dunn AK, Andermann ML, Ulbert I, Boas DA, Dale AM (2003) Coupling of total hemoglobin concentration, oxygenation, and neural activity in rat somatosensory cortex. Neuron 39:353-359.

Harris KD, Csicsvari J, Hirase H, Dragoi G, Buzsáki G (2003) Organization of cell assemblies in the hippocampus. Nature 424:552-556.

Krizhevsky A, Hinton G (2009) Learning multiple layers of features from tiny images.

Luczak A, Barthó P, Harris KD (2009) Spontaneous events outline the realm of possible sensory responses in neocortical populations. Neuron 62:413-425.

Luczak A, McNaughton BL, Kubo Y (2022) Neurons learn by predicting future activity. Nature Machine Intelligence (accepted).

Luczak A, Hackett TA, Kajikawa Y, Laubach M (2004) Multivariate receptive field mapping in marmoset auditory cortex. Journal of neuroscience methods 136:77-85.

McAdams HH, Shapiro L (1995) Circuit simulation of genetic networks. Science 269:650-656.

Tsodyks M, Kenet T, Grinvald A, Arieli A (1999) Linking spontaneous activity of single cortical neurons and the underlying functional architecture. Science 286:1943-1946.
